# *Enterococcus faecium* colonization and persistence in a model of diabetic wound infection

**DOI:** 10.1128/iai.00652-25

**Published:** 2026-04-30

**Authors:** Navin Jeyabalan, Frederick Reinhart Tanoto, Haris Antypas, Cheryl Jia Yi Neo, Rachel Jing Wen Tan, Kevin Pethe, David L. Becker, Claudia J. Stocks, Kimberly A. Kline

**Affiliations:** 1 Singapore Center for Environmental Life Sciences Engineering and School of Biological Sciences, Nanyang Technological University 54761, Singapore, Singapore; 2 Lee Kong Chian School of Medicine, Nanyang Technological University 54761, Singapore, Singapore; 3 Institute for Molecular Bioscience, The University of Queensland 1974, Brisbane, Australia; 4 Faculty of Medicine, University of Geneva 28535 , Geneva, Switzerland; University of Illinois Chicago, Chicago, Illinois, USA

**Keywords:** immune response, polymicrobial infection, *Staphylococcus epidermidis*, *Staphylococcus lentus*, biofilm infection, diabetes, *Enterococcus faecium*, mouse model

## Abstract

Chronic wound infections are a common comorbidity of diabetes mellitus and can progress to amputation if untreated, yet effective strategies to manage these infections are limited. Commensals such as *Enterococcus faecium* and *Staphylococcus epidermidis* can transition into opportunistic pathogens when host defenses are compromised, underscoring the complexity of chronic wound microbiology. *E. faecium*, particularly vancomycin-resistant strains, is an understudied, clinically important cause of chronic diabetic wound infections. Using a low-dose streptozocin-induced diabetic mouse model, we characterized *E. faecium* wound infection dynamics and identified differences in colonization and clearance compared to non-diabetic animals. At 8 h post-infection (hpi), control mice exhibited higher *E. faecium* wound colony-forming units (CFUs) than diabetic mice but cleared the infection more efficiently, resulting in similar CFU by 24 hpi. By contrast, diabetic mice showed impaired clearance, with elevated CFU persisting through 72 hpi. In mixed species infection with *S. epidermidis*, *S. epidermidis* CFU increased at 72 hpi, while *E. faecium* CFU remained comparable to single species infection. Despite strong initial cytokine and neutrophil responses, *E. faecium* persisted in all wounds. Sustained neutrophil recruitment at 72 hpi occurred only in diabetic mice, whereas macrophage accumulation increased from 24 to 72 hpi in all wounds, including sterile controls. Histological analysis showed epithelial hyper thickening in both groups, indicating that diabetes and *E. faecium* each contribute to impaired wound healing. This study establishes a diabetic mouse model of *E. faecium* wound infection and suggests that *E. faecium* modulates innate immune responses to persist in the wound bed.

## INTRODUCTION

Diabetes is a chronic metabolic disorder characterized by hyperglycemia resulting from partial or complete insulin insufficiency (1). The burden of diabetes in adults is increasing: the global number of adults with diabetes rose to about 828 million in 2022 from an estimated 198 million in 1990 (2). Individuals with diabetes are more susceptible to a range of infections, including urinary tract infections (UTIs), bacteremia, soft tissue infections, and cutaneous ulcerations, which, if left untreated, may progress to chronic wounds marked by persistent inflammation (3, 4). Chronic wound infections are defined as non-healing wounds that show no significant reduction in size after 2–4 weeks of medical care (5). These wound infections are often polymicrobial and pose a significant barrier to successful healing (5, 6). Reduced pain perception in individuals with diabetes increases the risk of neglecting minor cuts and abrasions (7), increasing the likelihood of infection and progression to chronic wound states. Of particular concern are diabetic foot ulcers (DFUs), which are difficult to treat, prone to infection, and frequently result in limb amputations (8). A population-based study in the UK reported that 40% of individuals with diabetes who also had peripheral vascular disease developed foot ulcerations (9). In Singapore, a qualitative study described the cost of managing diabetes-related amputation as up to $30,000 USD per patient (10).

DFUs are frequently dominated by opportunistic bacteria, including *Staphylococcus aureus*, *Pseudomonas aeruginosa*, *Streptococcus* spp., and Enterococci such as *Enterococcus faecalis* and *Enterococcus faecium* (11–13). Among these human-infection-associated Enterococci, most studies to date have focused on *E. faecalis*; however, the increasing global prevalence of antibiotic-resistant *E. faecium* demands urgent attention (14). Its clinical importance is reflected in its inclusion in the ESKAPE group, which describes six multidrug-resistant bacterial pathogens that are major threats to hospitalized individuals (15). *E. faecium* is also listed as a high-priority pathogen by both the WHO and CDC due to its resistance to last-line antibiotics and its role in healthcare-associated infections such as bacteremia and chronic wound infections (16, 17). In addition to its intrinsic resistance to last-line antibiotics such as vancomycin, daptomycin, and linezolid (18), *E. faecium* clinical isolates often encode virulence factors associated with biofilm formation (19), which can further complicate the treatment of enterococcal wound infections (20, 21). In a nursing facility, rectal swabs collected at the baseline visit revealed that 40% of patients were colonized with vancomycin-resistant *Enterococcus* species. Among the patients, 17.8% were colonized with vancomycin-resistant *E. faecium* and 8.4% with vancomycin-resistant *E. faecalis*, with some patients harboring both (22). Colonization with vancomycin-resistant *E. faecium* persisted for approximately twice as long as *E. faecalis* (22). However, experimental models to study infection dynamics of clinically relevant *E. faecium* in various niches remain scarce, highlighting the need in this study to characterize the infection dynamics and host response of vancomycin-resistant *E. faecium* strain E745 wound infection in a streptozotocin (STZ)-induced diabetic mouse model. We found that *E. faecium* successfully colonized both control and diabetic mice wounds, with differences in bacterial colonization and wound healing dynamics, as well as host immune and cytokine responses over the course of 72 h post-infection (hpi). Interestingly, in a co-infection model of murine wounds, *E. faecium* facilitated the growth of other *Staphylococcus* species unique to diabetic wounds, suggesting a possible role of *E. faecium*-mediated polymicrobial interactions during diabetic wound infections. Together, these findings are suggestive of a role for *E. faecium* in modulating skin commensals and delaying wound healing in diabetic wound infections.

## RESULTS

### *E. faecium* persists in diabetic wounds

**Fig 1:**
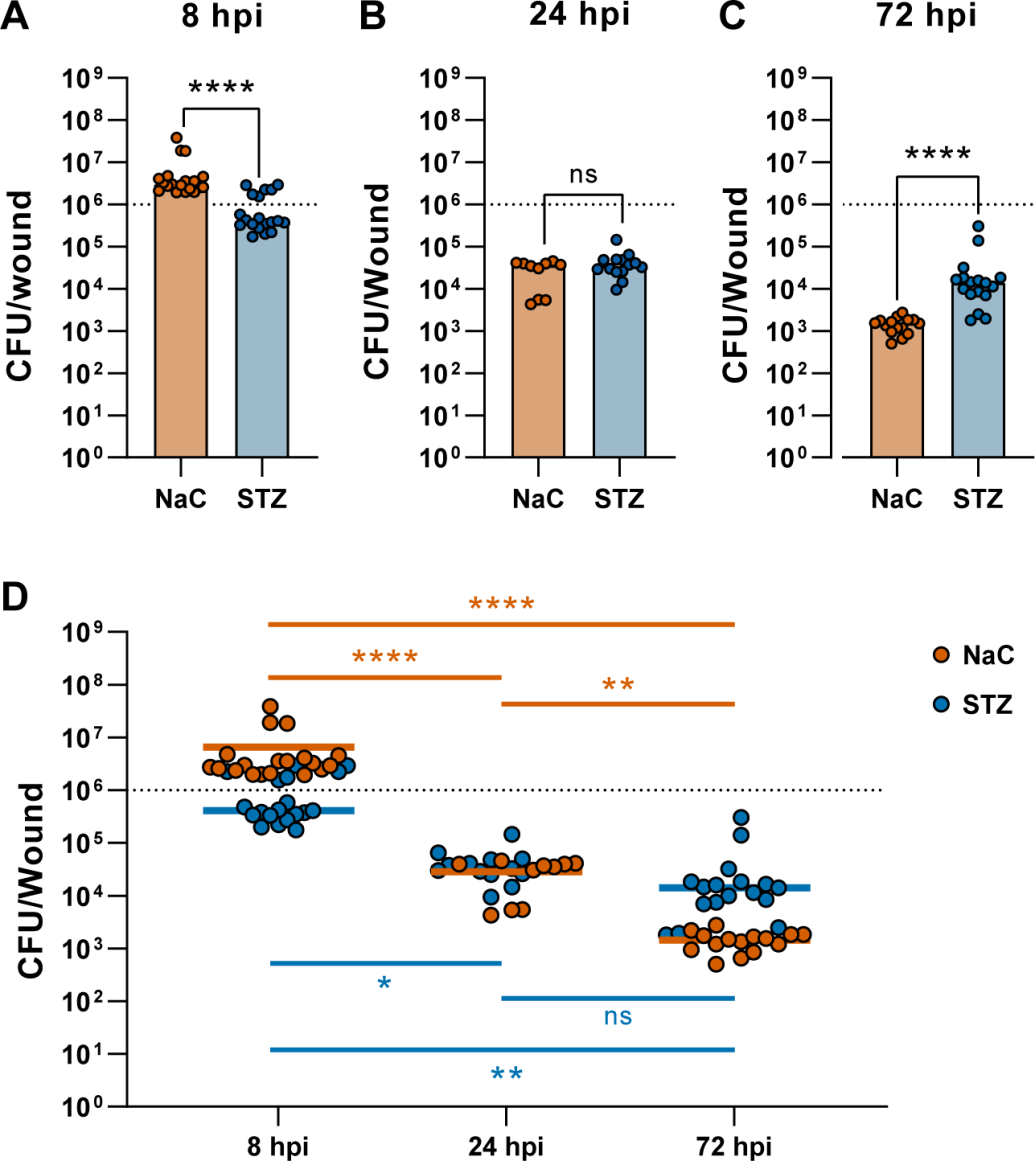
*E. faecium* persists at higher numbers at 72 hpi in diabetic mice. (**A–C**) CFU enumeration of control mice (NaC) and diabetic mice (STZ) wounds infected with *E. faecium* (10^6^ CFU) at (**A**) 8 hpi, (**B**) 24 hpi, and (**C**) 72 hpi. All CFU counts were enumerated on brain heart infusion + vancomycin (50 μg/mL). Bars represent median of *n* = 10–19 mice per group from *N* = 2–3. Statistical significance was determined using the Mann-Whitney test. ****P < 0.0001, ns = not significant. (**D**) Time course analysis of wound CFU counts from panels **A–C**. Statistical significance between different timepoints of the same infection group was assessed with two-way analysis of variance mixed model analysis. Dotted line represents *E. faecium* inoculum (10^6^). *P < 0.05, **P < 0.01, and ****P < 0.0001, ns non-significant.

To model *E. faecium* diabetic wound infection, C57BL/6J mice were rendered hyperglycemic with streptozotocin (STZ) (23). All control mice remained below the diabetic threshold of 300 mg/dL of blood glucose, whereas STZ-treated mice were diabetic by day 15 (Fig. S1A and B). Full-thickness, dorsal excisional wounds were inoculated with 10^6^ vancomycin-resistant *E. faecium* strain E745, and wound colony-forming units (CFU) were quantified at defined timepoints. At 8 hpi, CFU in control wounds rose to ~10^7^, indicating acute replication, while CFU in diabetic wounds were equivalent to inoculum (Fig. 1A). By 24 hpi, wound CFU in both groups declined to ~5 × 10^5^ CFU (Fig. 1B). At 72 hpi, CFU in the control wounds dropped further to ~10^3^, whereas CFU in the diabetic wounds stabilized at ~10^4^ (Fig. 1C). Thus, bacterial numbers decreased gradually in control wounds from 8 to 72 hpi but only declined between 8 and 24 hpi in diabetic mice, remaining stable thereafter (Fig. 1D). In preliminary experiments extended to 5 days post-infection (120 hpi), wound CFU were ~10^3^ in both groups (Fig. S1). However, interpretation was limited due to 40% mortality in infected diabetic mice prior to this timepoint (Fig. S1). Taken together, these findings indicate that *E. faecium* undergoes acute replication in control wounds but not in diabetic wounds, where it instead persists at higher levels at later timepoints of infection.

### *E. faecium* co-infection may facilitate the expansion of other opportunistic pathogens such as *S. epidermidis*

**Fig 2:**
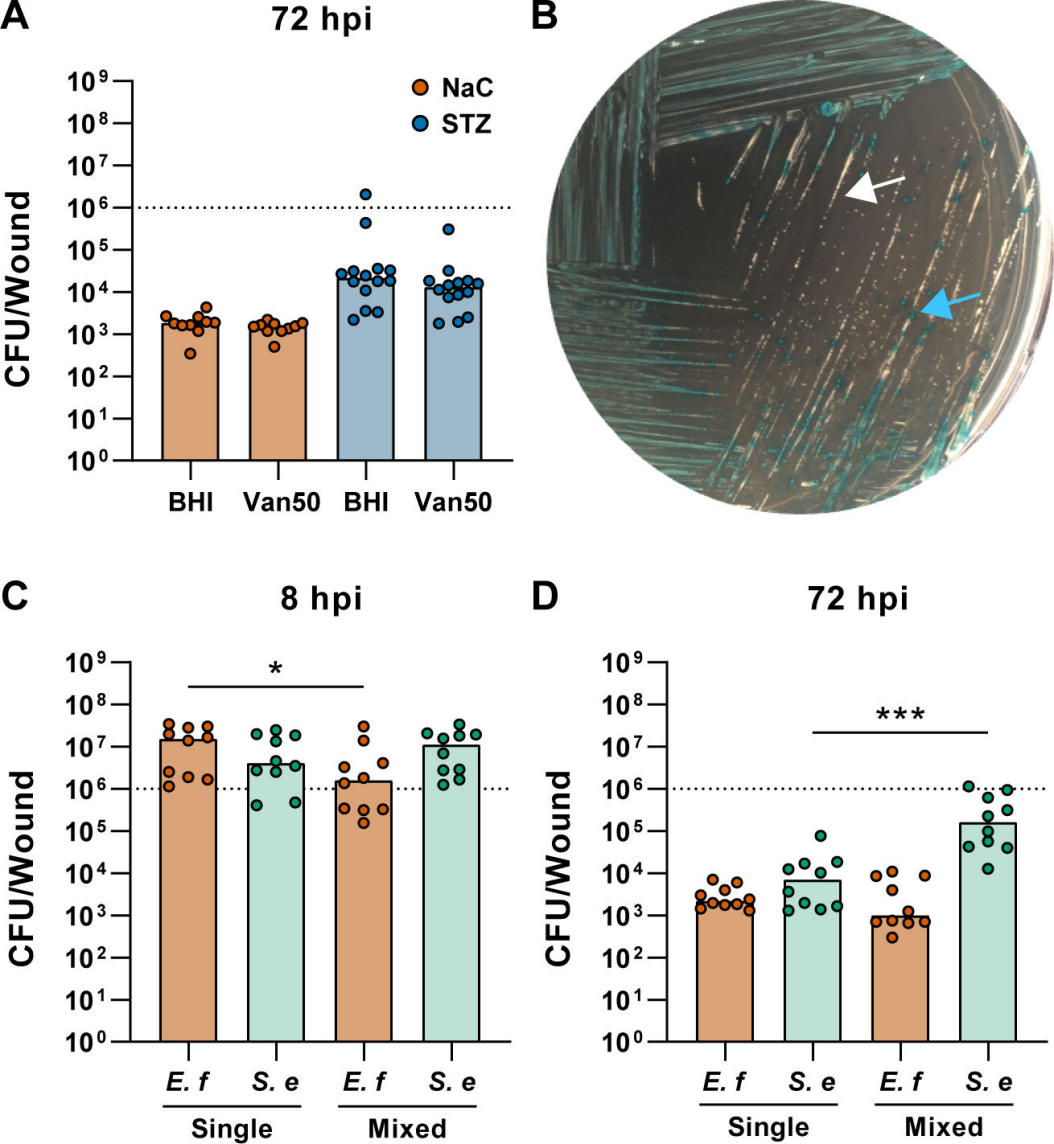
Mixed species wound infections enhance *Staphylococcus epidermidis* growth. (**A**) Wound CFU at 72 hpi from control (NaC) and diabetic (STZ) wounds plated on non-selective BHI and BHI + vancomycin (50 μg/mL; Van50) agars. Bars represent the median from *n* = 10–14 animals per infection group across two independent experiments. (**B**) UTI clarity streak plate from diabetic wound homogenates. Blue colonies (blue arrow) = *E. faecium*, white colonies (white arrow) = *S. lentus*. The plate image is representative of observations across three independent experiments. (**C and D**) Mixed species infection of control wounds with 1:1 *E. faecium* (*E.f*) and *S. epidermidis* (*S.e*; 10^6^ each). Wound CFU from (**C**) 8 hpi and (**D**) 72 hpi were quantified on BHI and Van50 agars. *S. lentus* CFU were defined as the difference between total BHI counts and *E. faecium*-specific Van50 CFU. Bars represent the median from *n* = 10 per infection group across two independent experiments. Statistical significance between BHI and vancomycin plate counts at each timepoint (**A**) or species-wise single and mixed species infection comparisons was determined by Mann-Whitney test. *P < 0.05, **P < 0.01, ***P < 0.001, and ****P < 0.0001; ns, non-significant.

During CFU enumeration, we plated wound homogenates on both selective (brain heart infusion [BHI] + 50 µg/mL vancomycin) and non-selective BHI agar. While CFUs were comparable on both media at 24 hpi (Fig. S2A), by 72 hpi, higher numbers were recovered on non-selective agar (Fig. 2A), suggesting the presence of a co-colonizing species. Plating on chromogenic agar confirmed the presence of both *Enterococcus* and *Staphylococcus* (Fig. 2B). The presence of *Staphylococcus* was unique to diabetic wounds at 72 h, regardless of infection status (Fig. S2B). 16S rRNA sequencing identified the co-colonizer to be *Staphylococcus lentus*, a murine skin commensal with pathogenic potential in animals that is rarely implicated in human infection (24–26). Thus, diabetic wounds uniquely supported colonization by the commensal *S. lentus*.

To test whether *S. lentus* influenced *E. faecium* infection, we compared single and mixed infections in control mice. Although there was a significant augmentation of *S. lentus* CFU at 8 hpi in mixed species infection, there was no significant difference at 72 hpi, during which staphylococcal co-colonization was observed (Fig. S2B through D). Meanwhile, *E. faecium* CFU was significantly lower in mixed species infection at 8 hpi but was comparable in both infections at 72 hpi. We next repeated the experiment with *Staphylococcus epidermidis*, a closely related human skin commensal and opportunistic pathogen (27, 28). In this case, we observed similar *S. epidermidis* CFU at 8 hpi, while *E. faecium* CFU was significantly lower (Fig. 2C). However, at 72 hpi, *S. epidermidis* CFUs were significantly higher in mixed compared to single species infection, whereas *E. faecium* CFUs were comparable in both infections (Fig. 2D). These data suggest that *E. faecium* co-infection may facilitate the expansion of other opportunistic pathogens such as *S. epidermidis* at later infection timepoints in the wound environment.

### Sustained neutrophil, but not macrophage, infiltration in *E. faecium-*infected diabetic wounds

**Fig 3:**
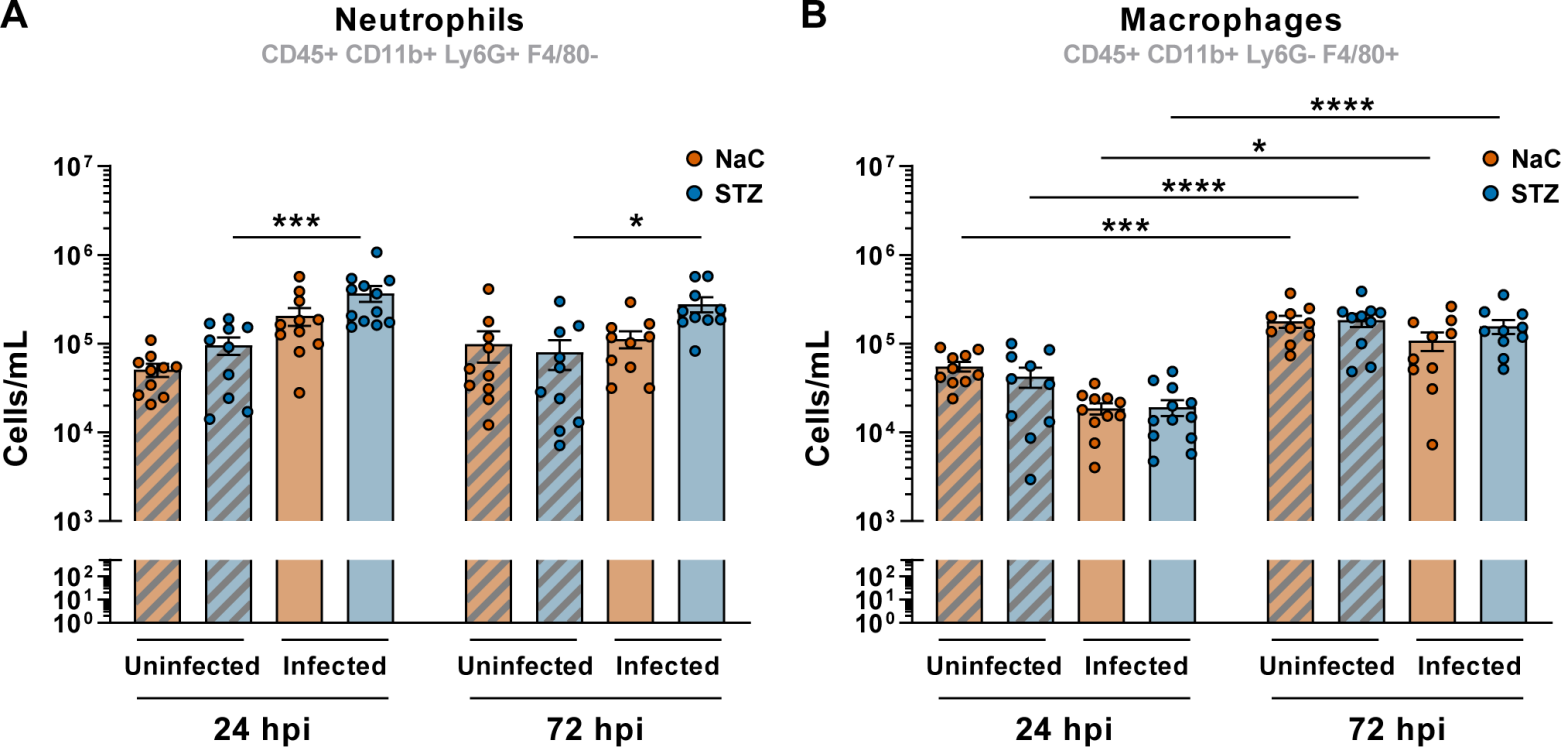
*E. faecium* infection elevates neutrophil infiltration in diabetic wounds, with macrophages elevated in 72 h wounds regardless of infection. Quantification of (**A**) neutrophil and (**B**) macrophage cell numbers from single cells dissociated from 24 and 72 hpi wounds. Bars indicated represent means ± SEM from *n* = 10 per infection group across two independent experiments. Statistical significance was determined by a three-way analysis of variance with Šídák’s multiple comparisons test. *P < 0.05, ***P < 0.001, and ****P < 0.0001.

We hypothesized that differences in *E. faecium* clearance between diabetic and control mice might reflect differences in the innate immune response. To test this, we profiled wound infiltrates by flow cytometry. Single-cell suspensions were stained and gated into neutrophil (CD11b^+^ Ly6G^+^ F4/80^−^) and macrophage (CD11b^+^ Ly6G^−^ F4/80^+^) populations (Fig. S3), and absolute cell numbers were quantified. At 24 h, we observed significantly more neutrophils in infected versus sterile diabetic wounds (Fig. 3A). We observed a similar trend in control mice, and overall neutrophil numbers were elevated in diabetic wounds in both conditions, albeit not statistically significant. At 72 h, although neutrophil infiltration was comparable in sterile and infected control mice, neutrophil infiltration was significantly higher in infected compared to sterile diabetic wounds, consistent with higher *E. faecium* CFU in diabetic wounds at this timepoint (Fig. 1C). The increase in neutrophil numbers at 24 hpi could not be explained by greater overall CD45^+^ immune cell infiltration, which was comparable across all populations by 72 hpi, suggesting that a greater proportion of neutrophils is present in 72 hpi diabetic wounds (Fig. S4A).

By contrast, macrophage numbers were comparable across all groups at 24 h (Fig. 3B). By 72 h, macrophage numbers had significantly increased relative to 24 h in both control and diabetic mice, independent of infection status (Fig. 3B), suggesting that wounding alone was sufficient to drive macrophage recruitment. Accordingly, CD45^+^ immune cell numbers, including neutrophils and macrophages, were consistently higher in wounded tissues compared to naïve unwounded skin (Fig. S4B, compared with Fig. 3; Fig. S4A). Minor differences were noted in baseline immune cell levels between diabetic and control mice, but these were not statistically significant. Together, these findings show that while macrophage recruitment is largely wound-driven, *E. faecium* infection promotes sustained neutrophil infiltration in diabetic wounds.

### *E. faecium* wound infection induces transient pro-inflammatory cytokine production

**Fig 4:**
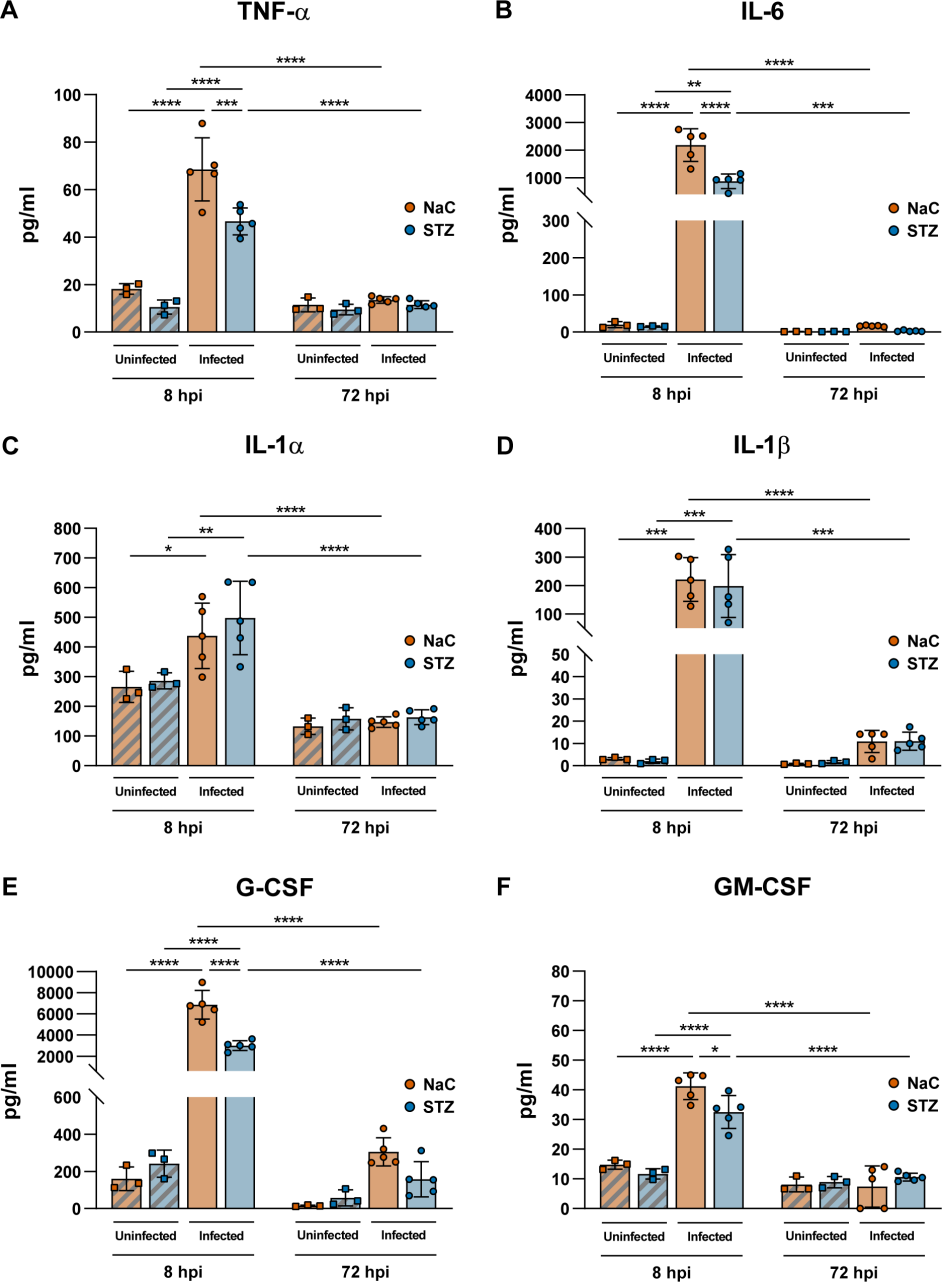
*E. faecium* wound infection triggers an acute inflammatory cytokine response that resolves by 72 h. Cytokine 23-plex assay of (**A**) TNF, (**B**) IL-6, (**C**) IL-1α, (**D**) IL-1β, (**E**) Granulocyte-Colony Stimulating Factor (G-CSF), and (**F**) Granulocyte, Monocyte-Colony Stimulating Factor (GM-CSF) in various wounds at 8 hpi and 72 hpi. Bars represent mean ± SD in *n* = 3–5 per infection group from one independent experiment. Statistical significance was determined by three-way analysis of variance with Šídák’s multiple comparisons test. *P < 0.05, **P < 0.01, ***P < 0.001, and ****P < 0.0001. Only meaningful comparisons with *P* < 0.05 are annotated.

We have previously shown that *E. faecalis* modulates host cytokine levels during wound infection (29), but the impact of *E. faecium*, particularly in a diabetic context, has not been investigated. We therefore measured pro-inflammatory cytokines and chemokines in wound lysates at 8 and 72 hpi (Fig. 4; Fig. S5). At 8 hpi, *E. faecium* induced robust TNF-α and IL-6, although levels were significantly lower in diabetic mice (Fig. 4A and B). IL-1α and IL-1β were similarly induced in response to infection in both control and diabetic mice (Fig. 4C and D). However, IL-1α was significantly induced only in diabetic mice (Fig. 4C and D). Granulocyte-Colony Stimulating Factor and Granulocyte, Monocyte-Colony Stimulating Factor were present at higher levels in response to infection, with both cytokine levels being significantly lower in infected diabetic mice compared to control (Fig. 4E and F). The anti-inflammatory cytokine IL-10 was comparably elevated in both control and diabetic groups in response to infection (Fig. S5A), whereas IL-17 and IFN-γ were elevated in infection, although they were present at lower levels in diabetic mice (Fig. S5B through D). Similarly, chemokines such as MCP-1 and MIP-1α were also increased in response to infection at 8 h, and MCP-1 was present at significantly lower levels in diabetic wounds (Fig. S5D, E and I). By 72 hpi, the levels of these cytokines were not significantly different across all four experimental groups, with generally comparable or lower levels compared to 8 hpi timepoints, particularly for infected groups (Fig. 4; Fig. S5), similar to the trend seen for *E. faecalis* wound infection (29). These findings show that while *E. faecium* induces a broad cytokine and chemokine response during acute infection, these responses are generally blunted in diabetic mice.

### *E. faecium* infection delays wound healing in diabetic tissues

**Fig 5:**
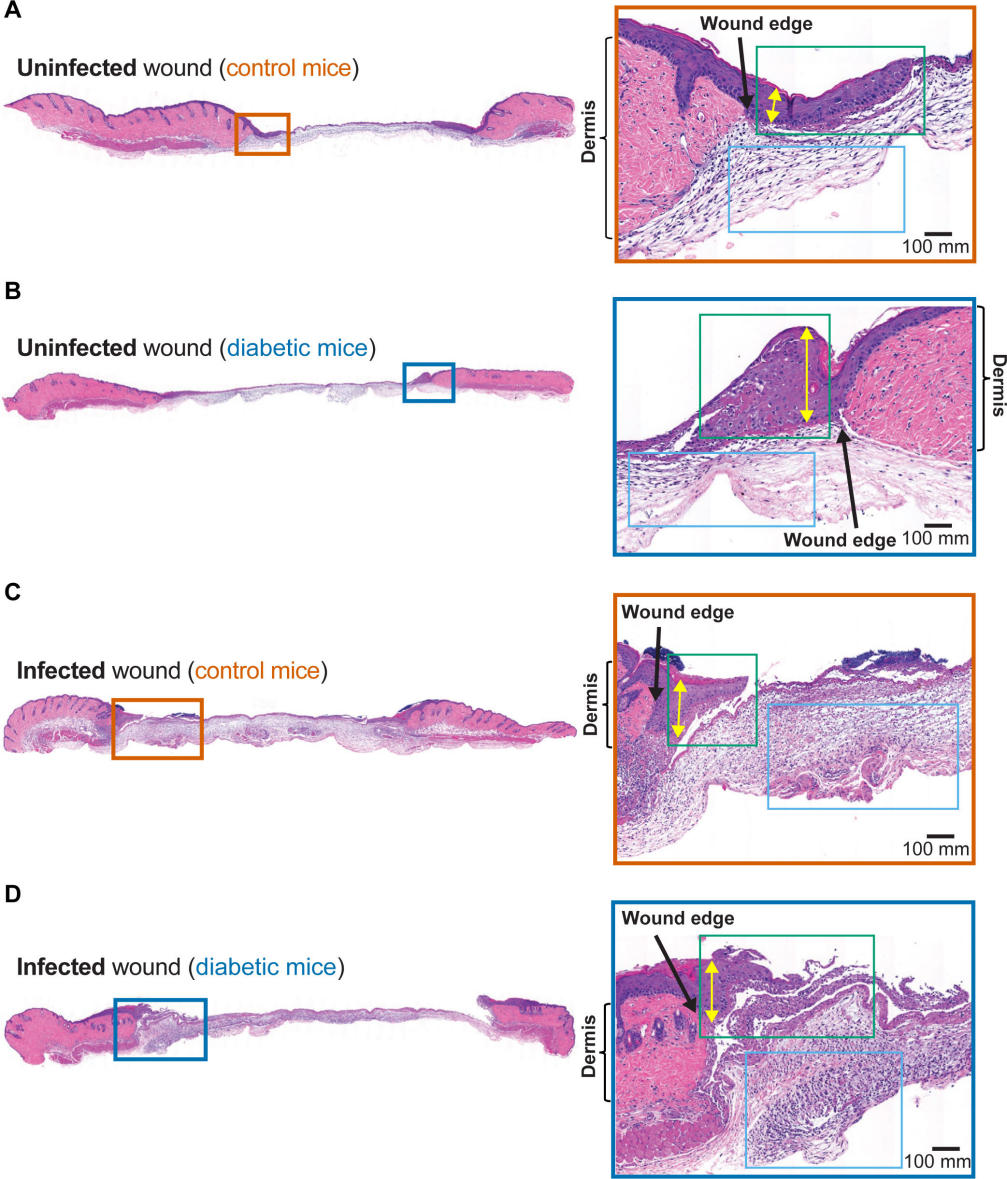
*E. faecium* infected and diabetic wounds exhibit epithelial hyper thickening with evidence of immune cell infiltration during infection. (**A–D**) Hematoxylin and eosin staining of 5 mm sections from (**A**) uninfected-control, (**B**) uninfected-diabetic, (**C**) infected-control, and (**D**) infected-diabetic wounds at 72 hpi. The right insets show a magnified view around the respective wound edges (black arrows). Yellow double arrows: epithelial thickness and green bordered regions: nascent epithelial growth. Light blue boxes represent regions representing immune cell infiltration. *n* = 2 per group, and sections displayed are representative of tissues sampled.

Macroscopic and histological examination of wounds can provide insights into wound healing during infection. *E. faecalis* can impede wound healing in control mice, with epidermal hyperplasia or hyper-thickened epidermis serving as a marker of impaired healing (29). To assess whether *E. faecium* impacts healing in a diabetic context, we performed hematoxylin and eosin (H&E) staining and macroscopic examination at 72 h across four groups: uninfected control and diabetic wounds (Fig. 5A and B) and *E. faecium* infected control and diabetic wounds (Fig. 5C and D). Wounds from uninfected control (Fig. 5A) and diabetic (Fig. 5B) mice exhibited re-epithelization, with new epidermis advancing from the wound edges (region indicated by green boxes, Fig. 5A and B). Uninfected diabetic wounds also showed a hyper-thickened epidermis, indicating impaired healing even in the absence of infection (epidermal thickness indicated by the yellow double arrow, Fig. 5B). In *E. faecium*-infected wounds, control mice similarly exhibited a hyper-thickened epidermis compared with adjacent old epidermis (indicated by the yellow double arrows, Fig. 5C and D). Furthermore, regions presenting dense infiltrates of small, round nucleated cells beneath the wound bed suggest actively infiltrating immune cells in response to infection (indicated by light blue boxes, Fig. 5C and D). Diabetic infected wounds exhibited equally pronounced hyper thickening and immune infiltration (Fig. 5D). Thus, both the diabetic environment and *E. faecium* infection appear to impact the progression of wound healing. These findings demonstrate that both diabetes and *E. faecium* infection independently impair wound healing, with overlapping pathological features including epidermal hyper thickening and excessive immune infiltration.

## DISCUSSION

The global rise in diabetes, coupled with the emergence of multidrug-resistant *E. faecium*, underscores an urgent need to understand the dynamics of *E. faecium* wound infection in diabetic populations (8, 11, 14, 30). Here, we show that diabetic wounds do not support acute replication of *E. faecium* after inoculation but present hindered clearance by 72 hpi. This finding suggests that diabetes-associated changes in the wound environment, potentially involving immune dysfunction or altered skin microbiota, constrain early bacterial expansion but permit longer-term persistence.

Although diabetes is classically associated with a pro-inflammatory baseline, marked by elevated plasma cytokines (31), our data reveal that the initial cytokine response to wound infection is blunted in diabetic mice. Despite this dampened cytokine response, *E. faecium* persists in diabetic wounds alongside sustained neutrophil infiltration, which is not mirrored by elevated cytokine levels or increased macrophage recruitment at later timepoints of infection. Our data also show that nearly every cytokine in diabetic infected wounds at 8 hpi was significantly lower compared to non-diabetic counterparts. It is unknown whether this stunted cytokine response is attributable to the lower bacterial burden at 8 hpi in diabetic infected wounds, or to an already compromised host immune response in the diabetic background. Supporting the latter hypothesis, the coexistence of persistent *E. faecium* titers and the sustained presence of neutrophils suggests a possible functional impairment of neutrophils in diabetic wounds, consistent with literature describing altered NETosis and phagocyte dysfunction in diabetes (32–34).

Microbial factors can further drive immune dysregulation. Previous studies have shown that *E. faecalis*, which is also commonly associated with human disease, can suppress elements of innate immunity. For instance, *E. faecalis* persists in wounds via a combination of reduced NF-κB signaling in macrophages, reduced phagocyte degranulation or phagolysosome formation, and replication within macrophages and neutrophils (29, 35–39). *E. faecalis* also impacts wound healing via impairment of epithelial to mesenchymal transition, a process essential for wound remodeling and re-epithelialization, even in non-diabetic wounds, providing precedence for direct modulation of wound healing mechanisms (40). Additionally, *E. faecalis*-derived reactive oxygen species have been linked to impaired wound healing (41). These immune- and tissue-modulatory effects likely extend to *E. faecium*, permitting both it and possible opportunistic partners to persist in the otherwise hostile wound microenvironment.

Polymicrobial interactions are a defining feature of chronic wounds, particularly in diabetes (19, 42, 43). This study identified commensal *S. lentus* as a co-colonizer exclusive to diabetic wounds, suggesting that diabetic host microenvironmental changes may favor colonization by commensal or opportunistic skin species. While *S. lentus* did not impact *E. faecium* persistence, mixed infections with *S. epidermidis*, a human skin commensal, led to augmented growth of the latter, suggesting that *E. faecium* may facilitate opportunist expansion through niche modification or immune modulation. This finding parallels reports of increased staphylococcal density and reduced overall diversity in diabetic mice skin (44–47). While preliminary co-infection experiments presented in this study are limited to non-diabetic mice, subsequent investigation of co-infection phenotypes with *E. faecium* in diabetic mice should be considered, as well as with other co-occurring species in diabetic wounds. Analogous polymicrobial occurrences were reported in group B streptococci diabetic wound infections, where *E. faecalis* and *Staphylococcus xylosus* were recovered from wound homogenates, underscoring both the importance of using non-selective media when isolating bacteria from diabetic hosts and the need to consider polymicrobial synergy in these infection settings (48). Although there is no evidence from previous literature that *E. faecium* can modulate the growth of co-infecting species during wound infection, there is precedence from studies in which *E. faecalis* augments *Escherichia coli* CFU in wounds (49) or *E. faecalis* growth is enhanced by *Staphylococcus aureus* (37)

Diabetic wounds, on the other hand, display hyper-thickened epidermis regardless of the infection status, indicating the diabetic environment may be the primary driver for delayed wound healing (50, 51). This reinforces the concept that diabetes and bacterial infection independently—and likely synergistically—impair core regenerative processes, an outcome exacerbated in polymicrobial contexts by biofilm formation and cooperative immune evasion strategies.

In conclusion, this work reveals distinct host-pathogen dynamics in diabetic wound infections, where *E. faecium* persists despite robust initial immune responses, contributing to delayed wound healing independent of host diabetic status. Further work should dissect the molecular mechanisms underlying immune modulation and interspecies interactions to inform targeted therapeutic strategies for chronic diabetic wound infections colonized by *E. faecium*.

## MATERIALS AND METHODS

### Bacterial strains and growth conditions

Vancomycin-resistant *Enterococcus faecium* strain E745 was grown overnight at 37°C in BHI broth (Neogen, Lansing, Michigan) without antibiotics. For mixed species experiments, *Staphylococcus epidermidis* strain ATCC 12228 and *Staphylococcus lentus* biological isolates from the mouse model of diabetic wound infections were grown overnight at 37°C in BHI broth without antibiotics. For wound infection experiments, overnight cultures of bacteria were washed and resuspended in PBS (Invitrogen, USA) to an OD of 0.4 (equivalent to 1 × 10^8^ CFU/mL) for infection.

### Streptozotocin-induced diabetic mice model

Diabetes was induced in male C57BL/6J mice (5–6 weeks old) via intraperitoneal injection of freshly prepared streptozotocin (STZ; Sigma, USA, Cat# S0130-50MG), administered at 50 mg/kg in 0.1 M sodium citrate solution (NaC; Sigma, USA, Cat# C7254-1KG), pH 4.5, daily for 5 consecutive days (23). Control mice received only an equivalent NaC solution, pH 4.5. Mice were fasted 4–5 h prior to injection and left on a regular diet for 7 days post-injection before being deemed ready for infection. Diabetes was confirmed 7 days post-injection by fasting blood glucose measurements (>300 mg/dL) using an ACCU-CHEK Instant Glucometer (Model 961; Roche International).

### Murine excisional wound infection model

Excisional wounds (6 mm) were created on the dorsal skin of anesthetized 7–8 week-old C57BL/6J male mice (isoflurane), as previously described (29). Hair was removed by shaving and depilatory cream, and skin was disinfected with ethanol, prior to wounding. Wounds were inoculated with 10^6^ cells of *E. faecium* (E745) either alone or in combination (1:1) with *S. epidermidis* or *S. lentus* (10^6^ cells/species). Tegaderm dressing (3M, St Paul Minnesota, USA) was applied, and mice were monitored regularly prior to euthanization at 8, 24, or 72 hpi.

### CFU enumeration from wound homogenates

Excised wounds (including Tegaderm) were homogenized in 1 mL PBS using a Lysing Matrix M (MP Biomedicals, USA, Cat# 6923050) at 4.0 m/s for 20 s for five rounds. Wound homogenates were serially diluted in PBS, and serial dilutions were plated on BHI agar with or without 50 μg/mL of vancomycin (Van50; Merck, USA, Cat# 1709007) for CFU enumeration. The vancomycin-resistant *E. faecium* CFU counts were taken from Van50 plates alone, while vancomycin-susceptible *S. epidermidis* and *S. lentus* CFU counts were taken as the difference between total BHI counts and *E. faecium*-specific Van50 counts. Wound homogenates were also plated on UTI Clarity agar (Thermo Fisher Scientific, Brilliance UTI Clarity Agar, USA, Cat# CM1106T) to identify contaminants or common co-colonizers of enterococcal infections.

### Cytokine analysis

After homogenization as above, wound lysates were diluted 1:2 and analyzed for total cytokine concentrations using the Bio-Plex PRO Mouse Cytokine 23-plex Assay (Bio-Rad, California, USA), following manufacturer’s instructions as previously described (52).

### Histology

Excised wound tissues, from a total of two mice per group (a total of eight tissues), were bisected (Tegaderm removed) and fixed in 4% paraformaldehyde (PFA; Sigma, USA) for 24 h at 4°C. Fixed tissues were embedded in paraffin, sectioned at 5 mm thickness, stained with hematoxylin and eosin (H&E), and imaged with the help of Advanced Molecular Pathology Laboratory at the Institute of Molecular and Cell Biology, A*STAR, Singapore. Slide-scanned images were analyzed via the Carl Zeiss Zen Lite software.

### Flow cytometry

Excised wound tissues were digested with 0.2 mg/mL Liberase TL (Merck, USA) at 37°C for 1 h, and single-cell suspensions were filtered through a 40 μm cell strainer (SPF Life Sciences, South Korea). The single-cell suspension was normalized to a cell count of 1 × 10^7^ cells/mL. A total of 50 μL of the suspension was blocked with 1:100 TruStain FcX PLUS anti-mouse CD16/32 (BioLegend, USA, Cat# Cat# 156604) antibodies for 30 min on ice and then immediately stained for 1 h on ice with the following antibodies, each at 1:100 dilution: BV510 anti-mouse CD45 (BioLegend, Cat# 103138, USA), FITC anti-mouse Ly6G (BioLegend, Cat# 127606, USA), APC anti-mouse F4/80 (BioLegend, Cat# 123116, USA), and PE anti-mouse/human CD11b (BioLegend, Cat# 101208, USA). After fixation with 4% PFA, samples were analyzed using a 5-laser BD LSRFortessa X-20.

For absolute counts of immune cell infiltrates, AccuCheck Counting Beads (Invitrogen, Cat# PCB100, USA) were added immediately before flow cytometry in a ratio of 100 μL beads to 400 μL of single-cell suspensions, according to manufacturer protocols. Absolute counts were calculated by normalizing the total number of cells to the total number of beads acquired (Fig S3).

### Data and statistical analysis

Flow cytometry data were plotted and analyzed using FlowJo v10.8.0 (Becton Dickinson, USA). Other graphs and statistical analysis were performed with GraphPad Prism (Version 10.01 for Macintosh OS). Details on specific tests applied and data obtained are contained within figure legends.

## Data Availability

We will make data fully available and without restriction, upon request.

## SUPPLEMENTAL MATERIAL

Supplemental Figures iai.00652-25-s0001.docx Figures S1 to S5.

ASM does not own the copyrights to Supplemental Material that may be linked to, or accessed through, an article. The authors have granted ASM a non-exclusive, world-wide license to publish the Supplemental Material files. Please contact the corresponding author directly for reuse.

## References

[1] Egan AM, Dinneen SF. 2019. What is diabetes? Medicine (Abingdon) 47:1–4. doi:10.1016/j.mpmed.2018.10.002

[2] Zhou B, Rayner AW, Gregg EW, Sheffer KE, Carrillo-Larco RM, Bennett JE, Shaw JE, Paciorek CJ, Singleton RK, Barradas Pires A, et al.. 2024. Worldwide trends in diabetes prevalence and treatment from 1990 to 2022: a pooled analysis of 1108 population-representative studies with 141 million participants. Lancet 404:2077–2093. doi:10.1016/S0140-6736(24)02317-1 39549716 PMC7616842

[3] Pearson-Stuttard J, Blundell S, Harris T, Cook DG, Critchley J. 2016. Diabetes and infection: assessing the association with glycaemic control in population-based studies. Lancet Diabetes Endocrinol 4:148–158. doi:10.1016/S2213-8587(15)00379-4 26656292

[4] Joshi N, Caputo GM, Weitekamp MR, Karchmer AW. 1999. Infections in patients with diabetes mellitus. N Engl J Med 341:1906–1912. doi:10.1056/NEJM199912163412507 10601511

[5] Siddiqui AR, Bernstein JM. 2010. Chronic wound infection: facts and controversies. Clin Dermatol 28:519–526. doi:10.1016/j.clindermatol.2010.03.009 20797512

[6] Sen CK. 2021. Human wound and its burden: updated 2020 compendium of estimates. Adv Wound Care 10:281–292. doi:10.1089/wound.2021.0026 PMC8024242 33733885

[7] Corbett C, Jolley J, Barson E, Wraight P, Perrin B, Fisher C. 2019. Cognition and understanding of neuropathy of inpatients admitted to a specialized tertiary diabetic foot unit with diabetes-related foot ulcers. Int J Low Extrem Wounds 18:294–300. doi:10.1177/1534734619862085 31307246

[8] Jeffcoate WJ, Harding KG. 2003. Diabetic foot ulcers. Lancet 361:1545–1551. doi:10.1016/S0140-6736(03)13169-8 12737879

[9] Kumar S, Ashe HA, Parnell LN, Fernando DJS, Tsigos C, Young RJ, Ward JD, Boulton AJM. 1994. The prevalence of foot ulceration and its correlates in type 2 diabetic patients: a population-based study. Diabet Med 11:480–484. doi:10.1111/j.1464-5491.1994.tb00310.x 8088127

[10] Foong HF, Kyaw BM, Upton Z, Tudor Car L. 2020. Facilitators and barriers of using digital technology for the management of diabetic foot ulcers: a qualitative systematic review. Int Wound J 17:1266–1281. doi:10.1111/iwj.13396 32390305 PMC7948580

[11] Jneid J, Lavigne JP, La Scola B, Cassir N. 2017. The diabetic foot microbiota: a review. Hum Microbiome J 5–6:1–6. doi:10.1016/j.humic.2017.09.002

[12] Keogh RA, Huyvaert S, Moore GD, Horswill AR, Doran KS. 2024. Virulence characteristics of Gram-positive bacteria isolated from diabetic foot ulcers. FEMS Microbes 5:xtae013. doi:10.1093/femsmc/xtae013 38783991 PMC11114470

[13] Shettigar K, Bhat DV, Satyamoorthy K, Murali TS. 2018. Severity of drug resistance and co-existence of Enterococcus faecalis in diabetic foot ulcer infections. Folia Microbiol (Praha) 63:115–122. doi:10.1007/s12223-017-0547-2 28889401

[14] Guzman Prieto AM, van Schaik W, Rogers MRC, Coque TM, Baquero F, Corander J, Willems RJL. 2016. Global emergence and dissemination of enterococci as nosocomial pathogens: attack of the clones? Front Microbiol 7:788. doi:10.3389/fmicb.2016.00788 27303380 PMC4880559

[15] Miller WR, Arias CA. 2024. ESKAPE pathogens: antimicrobial resistance, epidemiology, clinical impact and therapeutics. Nat Rev Microbiol 22:598–616. doi:10.1038/s41579-024-01054-w 38831030 PMC13147291

[16] WHO. 2024. WHO bacterial priority pathogens list: updated 2024, p 10–13

[17] World Health Organization. 2024. WHO updates list of drug-resistant bacteria most threatening to human health

[18] Ramirez-Acuña JM, Cardenas-Cadena SA, Marquez-Salas PA, Garza-Veloz I, Perez-Favila A, Cid-Baez MA, Flores-Morales V, Martinez-Fierro ML. 2019. Diabetic foot ulcers: current advances in antimicrobial therapies and emerging treatments. Antibiotics (Basel) 8:193. doi:10.3390/antibiotics8040193 31652990 PMC6963879

[19] Dowd SE, Wolcott RD, Sun Y, McKeehan T, Smith E, Rhoads D. 2008. Polymicrobial nature of chronic diabetic foot ulcer biofilm infections determined using bacterial tag encoded FLX amplicon pyrosequencing (bTEFAP). PLoS One 3:e3326. doi:10.1371/journal.pone.0003326 18833331 PMC2556099

[20] Teng F, Kawalec M, Weinstock GM, Hryniewicz W, Murray BE. 2003. An Enterococcus faecium secreted antigen, SagA, exhibits broad-spectrum binding to extracellular matrix proteins and appears essential for E. faecium growth. Infect Immun 71:5033–5041. doi:10.1128/IAI.71.9.5033-5041.2003 12933846 PMC187350

[21] Paganelli FL, de Been M, Braat JC, Hoogenboezem T, Vink C, Bayjanov J, Rogers MRC, Huebner J, Bonten MJM, Willems RJL, Leavis HL. 2015. Distinct SagA from hospital-associated clade A1 Enterococcus faecium strains contributes to biofilm formation. Appl Environ Microbiol 81:6873–6882. doi:10.1128/AEM.01716-15 26209668 PMC4561713

[22] Davis E, Hicks L, Ali I, Salzman E, Wang J, Snitkin E, Gibson K, Cassone M, Mody L, Foxman B. 2020. Epidemiology of vancomycin-resistant Enterococcus faecium and Enterococcus faecalis colonization in nursing facilities. Open Forum Infect Dis 7:ofz553. doi:10.1093/ofid/ofz553 31993459 PMC6979485

[23] Furman BL. 2015. Streptozotocin-induced diabetic models in mice and rats. Curr Protoc Pharmacol 70:5. doi:10.1002/0471141755.ph0547s70 26331889

[24] Mazal C, Sieger B. 2010. Staphylococcus lentus: the troublemaker. Int J Infect Dis 14:e397. doi:10.1016/j.ijid.2010.02.502

[25] Szczuka E, Wesołowska M, Krawiec A, Kosicki JZ. 2023. Staphylococcal species composition in the skin microbiota of domestic pigeons (Columba livia domestica). PLoS One 18:e0287261. doi:10.1371/journal.pone.0287261 37436966 PMC10337865

[26] Hay CY, Sherris DA. 2020. Staphylococcus lentus sinusitis: a new sinonasal pathogen. Ear Nose Throat J 99:NP62–NP63. doi:10.1177/0145561319848990 31072191

[27] Otto M. 2009. Staphylococcus epidermidis — the 'accidental' pathogen. Nat Rev Microbiol 7:555–567. doi:10.1038/nrmicro2182 19609257 PMC2807625

[28] Brown MM, Horswill AR. 2020. Staphylococcus epidermidis—Skin friend or foe? PLOS Pathog 16:e1009026. doi:10.1371/journal.ppat.1009026 33180890 PMC7660545

[29] Chong KKL, Tay WH, Janela B, Yong AMH, Liew TH, Madden L, Keogh D, Barkham TMS, Ginhoux F, Becker DL, Kline KA. 2017. Enterococcus faecalis modulates immune activation and slows healing during wound infection. J Infect Dis 216:1644–1654. doi:10.1093/infdis/jix541 29045678 PMC5854026

[30] Hussain A. 2018. Diabetes in Asia: special challenges and solutions. J Diabetol 9:69. doi:10.4103/jod.jod_22_18

[31] Devaraj S, Dasu MR, Jialal I. 2010. Diabetes is a proinflammatory state: a translational perspective. Expert Rev Endocrinol Metab 5:19–28. doi:10.1586/eem.09.44 20204165 PMC2829992

[32] Farhan A, Hassan G, Ali SHL, Yousaf Z, Shafique K, Faisal A, Younis BB, Mirza S. 2023. Spontaneous NETosis in diabetes: a role of hyperglycemia mediated ROS and autophagy. Front Med (Lausanne) 10:1076690. doi:10.3389/fmed.2023.1076690 36895726 PMC9988915

[33] Thimmappa PY, Vasishta S, Ganesh K, Nair AS, Joshi MB. 2023. Neutrophil (dys)function due to altered immuno-metabolic axis in type 2 diabetes: implications in combating infections. Hum Cell 36:1265–1282. doi:10.1007/s13577-023-00905-7 37115481 PMC10284735

[34] Wong SL, Demers M, Martinod K, Gallant M, Wang Y, Goldfine AB, Kahn CR, Wagner DD. 2015. Diabetes primes neutrophils to undergo NETosis, which impairs wound healing. Nat Med 21:815–819. doi:10.1038/nm.3887 26076037 PMC4631120

[35] da Silva RAG, Tay WH, Ho FK, Tanoto FR, Chong KKL, Choo PY, Ludwig A, Kline KA. 2022. Enterococcus faecalis alters endo-lysosomal trafficking to replicate and persist within mammalian cells. PLoS Pathog 18:e1010434. doi:10.1371/journal.ppat.1010434 35390107 PMC9017951

[36] Tien BYQ, Goh HMS, Chong KKL, Bhaduri-Tagore S, Holec S, Dress R, Ginhoux F, Ingersoll MA, Williams RBH, Kline KA. 2017. Enterococcus faecalis promotes innate immune suppression and polymicrobial catheter-associated urinary tract infection. Infect Immun 85:e00378-17. doi:10.1128/IAI.00378-17 28893918 PMC5695114

[37] Kao PH-N, Ch’ng J-H, Chong KKL, Stocks CJ, Wong SL, Kline KA. 2023. Enterococcus faecalis suppresses Staphylococcus aureus-induced NETosis and promotes bacterial survival in polymicrobial infections. FEMS Microbes 4:xtad019. doi:10.1093/femsmc/xtad019 37900578 PMC10608956

[38] Stocks CJ, da Silva RAG, Antypas H, Jeyabalan N, Wong SL, Kline KA. 2026. Enterococcus faecalis persists and replicates intracellularly within neutrophils. Infect Immun 94:e0036425. doi:10.1128/iai.00364-25 41400490 PMC12797935

[39] Tanoto FR, Liew JH, Stocks CJ, Chong KKL, Pethe K, Antypas H, Kline KA. 2025. Gelatinase regulates the egress of intracellular replicating populations during Enterococcus faecalis infection. PLOS Pathogens. doi:10.1371/journal.ppat.1013738 PMC12994788 41805723

[40] Celik C, Lee STT, Tanoto FR, Veleba M, Kline K, Thibault G. 2024. Decoding the complexity of delayed wound healing following Enterococcus faecalis infection. eLife 13:RP95113. doi:10.7554/eLife.95113 38767331 PMC11105157

[41] Tan CAZ, Antypas H, Kline KA. 2020. Overcoming the challenge of establishing biofilms in vivo: a roadmap for Enterococci. Curr Opin Microbiol 53:9–18. doi:10.1016/j.mib.2020.01.013 32062025

[42] Wolcott RD, Rhoads DD, Bennett ME, Wolcott BM, Gogokhia L, Costerton JW, Dowd SE. 2010. Chronic wounds and the medical biofilm paradigm. J Wound Care 19:45–46. doi:10.12968/jowc.2010.19.2.46966 20216488

[43] Singh N, Armstrong DG, Lipsky BA. 2005. Preventing foot ulcers in patients with diabetes. JAMA 293:217–228. doi:10.1001/jama.293.2.217 15644549

[44] Weyrich LS, Dixit S, Farrer AG, Cooper AJ, Cooper AJ. 2015. The skin microbiome: associations between altered microbial communities and disease. Australas J Dermatol 56:268–274. doi:10.1111/ajd.12253 25715969

[45] Gontcharova V, Youn E, Sun Y, Wolcott RD, Dowd SE. 2010. A comparison of bacterial composition in diabetic ulcers and contralateral intact skin. Open Microbiol J 4:8–19. doi:10.2174/1874285801004010008 20461221 PMC2866239

[46] Price LB, Liu CM, Melendez JH, Frankel YM, Engelthaler D, Aziz M, Bowers J, Rattray R, Ravel J, Kingsley C, Keim PS, Lazarus GS, Zenilman JM. 2009. Community analysis of chronic wound bacteria using 16S rRNA gene-based pyrosequencing: impact of diabetes and antibiotics on chronic wound microbiota. PLoS One 4:e6462. doi:10.1371/journal.pone.0006462 19649281 PMC2714066

[47] Grice EA, Snitkin ES, Yockey LJ, Bermudez DM, Liechty KW, Segre JA, NISC Comparative Sequencing Program. 2010. Longitudinal shift in diabetic wound microbiota correlates with prolonged skin defense response. Proc Natl Acad Sci USA 107:14799–14804. doi:10.1073/pnas.1004204107 20668241 PMC2930465

[48] Keogh RA, Haeberle AL, Langouët-Astrié CJ, Kavanaugh JS, Schmidt EP, Moore GD, Horswill AR, Doran KS. 2022. Group B Streptococcus adaptation promotes survival in a hyperinflammatory diabetic wound environment. Sci Adv 8:eadd3221. doi:10.1126/sciadv.add3221 36367946 PMC9651866

[49] Keogh D, Tay WH, Ho YY, Dale JL, Chen S, Umashankar S, Williams RBH, Chen SL, Dunny GM, Kline KA. 2016. Enterococcal metabolite cues facilitate interspecies niche modulation and polymicrobial infection. Cell Host Microbe 20:493–503. doi:10.1016/j.chom.2016.09.004 27736645 PMC5076562

[50] Falanga V. 2005. Wound healing and its impairment in the diabetic foot. Lancet 366:1736–1743. doi:10.1016/S0140-6736(05)67700-8 16291068

[51] Zhao G, Usui ML, Underwood RA, Singh PK, James GA, Stewart PS, Fleckman P, Olerud JE. 2012. Time course study of delayed wound healing in a biofilm-challenged diabetic mouse model. Wound Repair Regen 20:342–352. doi:10.1111/j.1524-475X.2012.00793.x 22564229 PMC3349451

[52] Rousseau M, Goh HMS, Holec S, Albert ML, Williams RB, Ingersoll MA, Kline KA. 2016. Bladder catheterization increases susceptibility to infection that can be prevented by prophylactic antibiotic treatment. JCI Insight 1:e88178. doi:10.1172/jci.insight.88178 27699248 PMC5033754

