# Supplementary material for: Enterococcus faecium colonization and persistence in a model of diabetic wound infection
Source: Infect Immun. 2026 Apr 30;94(6):e00652-25. doi: 10.1128/iai.00652-25 (PMC13248726; doi:10.1128/iai.00652-25)
Supplement: Supplemental Figures — Figures S1 to S5. [file iai.00652-25-s0001.docx]

***Enterococcus faecium* colonization and persistence in a model of diabetic wound infection**

Navin Jeyabalan^a^, Frederick Reinhart Tanoto^a^, Haris Antypas^a^, Cheryl Neo Jia Yi^a^, Rachel Tan Jing Wen^a^, Kevin Pethe^a,b^, David L. Becker^b^, Claudia J. Stocks^a,c*^, Kimberly A. Kline^a,d*^

^a^ Singapore Centre for Environmental Life Sciences Engineering and School of Biological Sciences, Nanyang Technological University, Singapore.

^b^ Lee Kong Chian School of Medicine, Nanyang Technological University, Singapore

^c^ Institute for Molecular Bioscience, The University of Queensland, Brisbane, Australia

School of Biological Sciences, Nanyang Technological University, Singapore.

^d^ University of Geneva, Faculty of Medicine, Geneva, Switzerland.

Running Head: *Enterococcus faecium* diabetic wound infection model

Address correspondence to:

* Claudia J Stocks [c.stocks@uq.edu.au](mailto:c.stocks@uq.edu.au), Kimberly A Kline [kimberly.kline@unige.ch](mailto:kimberly.kline@unige.ch)


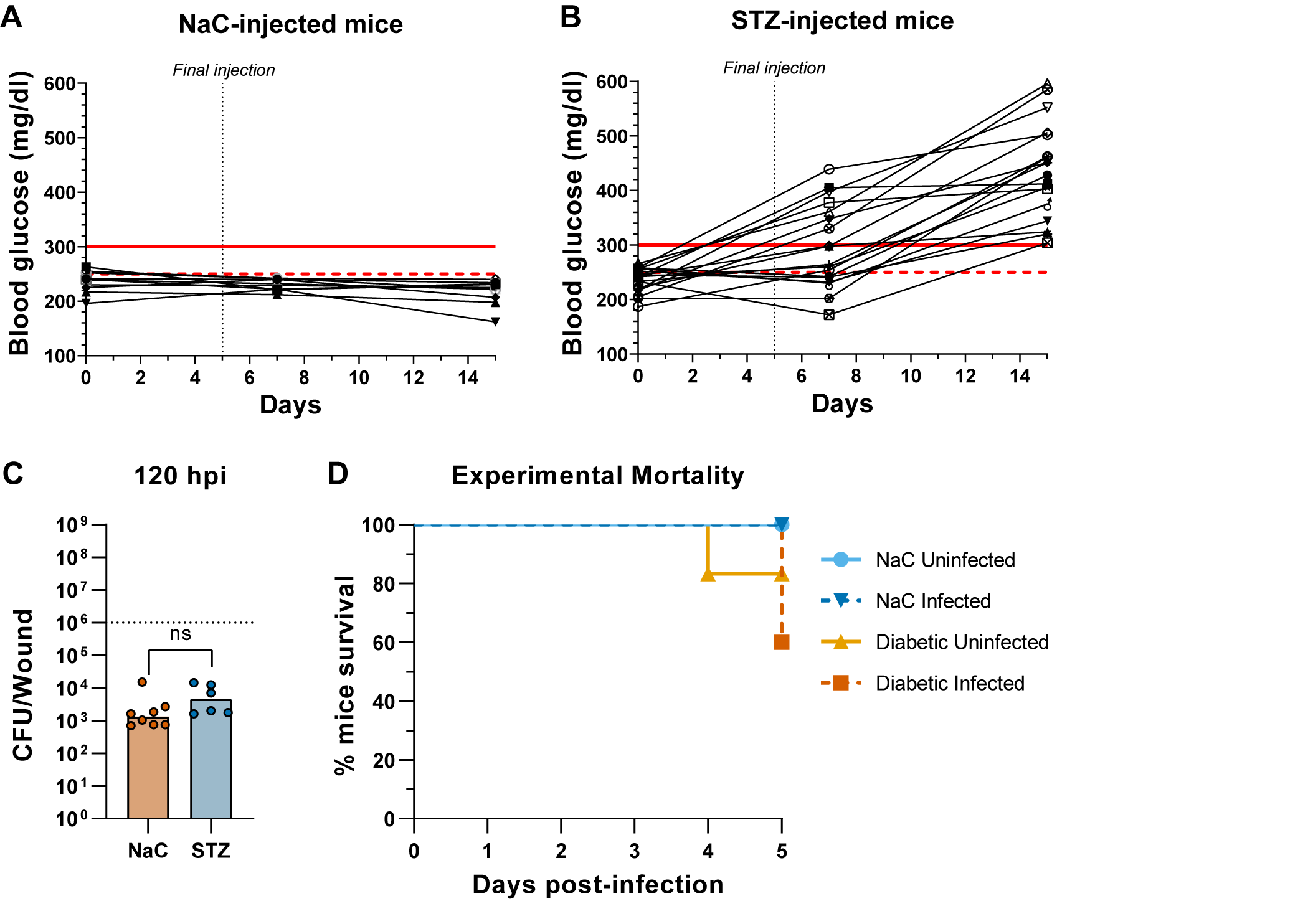


**Supplementary Fig. 1: STZ treatment induces hyperglycemia and increases mortality following wounding and infection. (A-B)** Fasted mice were injected with either **(A)** sodium citrate (NaC) or **(B)** streptozotocin (STZ) for 5 days, and monitored for fasting blood glucose concentrations at 0, 7 and 15 days. Each data point depicts matched blood glucose measurements from n=18 mice across 3 independent experiments. **(C)** Wound CFU of control (NaC) and diabetic (STZ) mice at 5 dpi on BHI + vancomycin (50 μg/ml). Bars represent median from n = 6-8 per infection group from one independent experiment. Dotted line represents *E. faecium* inoculum (10^6^). Statistical significance was determined by Mann-Whitney Test. ns = non-significant. **(D)** Mortality of mice up to 5 dpi from one independent experiment (n = 10 per mice group), showing percentage survival of control (NaC) mice or diabetic mice with or without *E. faecium* infection:


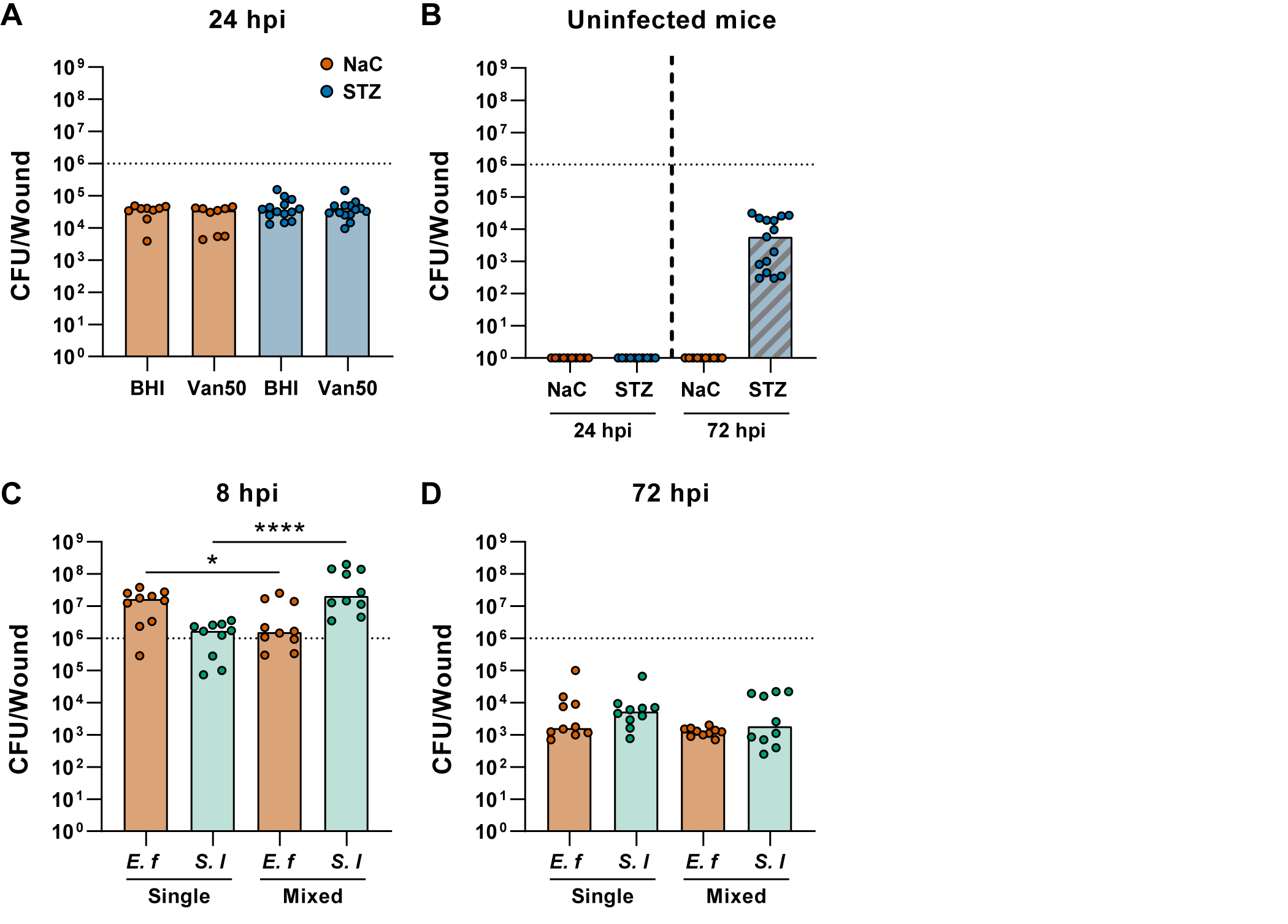


**Supplementary Fig 2: *S. lentus* uniquely colonizes diabetic wounds at 72 hpi without altering mixed species growth dynamics.** All wound homogenates were plated on plated on non-selective BHI and BHI + vancomycin (50 μg/ml) (Van50) agars. **(A-B)** Wound CFU from control (NaC) and diabetic (STZ) wounds after **(A)** 24 h of infection with *E. faecium*, or **(B**) 24 or 72 h of mock-infection with PBS. Bars represent median from n = 10-15 animals per infection group across 2-3 independent experiments. **(C-D)** Mixed species infection of NaC wounds with 1:1 *E. faecium* (*E.f)* and *S. lentus (S.l)* (10^6^ each). Wound CFU from **(C)** 8 hpi and **(D)** 72 hpi were quantified on BHI and Van50 agars. *S. lentus* CFU counts were taken as the difference between total BHI counts and *E. faecium*-specific Van50 counts. Bars represent median from n = 10 per infection group across 2 independent experiments. Statistical significance between single and mixed species infections was determined by Mann-Whitney Test. * p<0.05, **** p<0.0001.


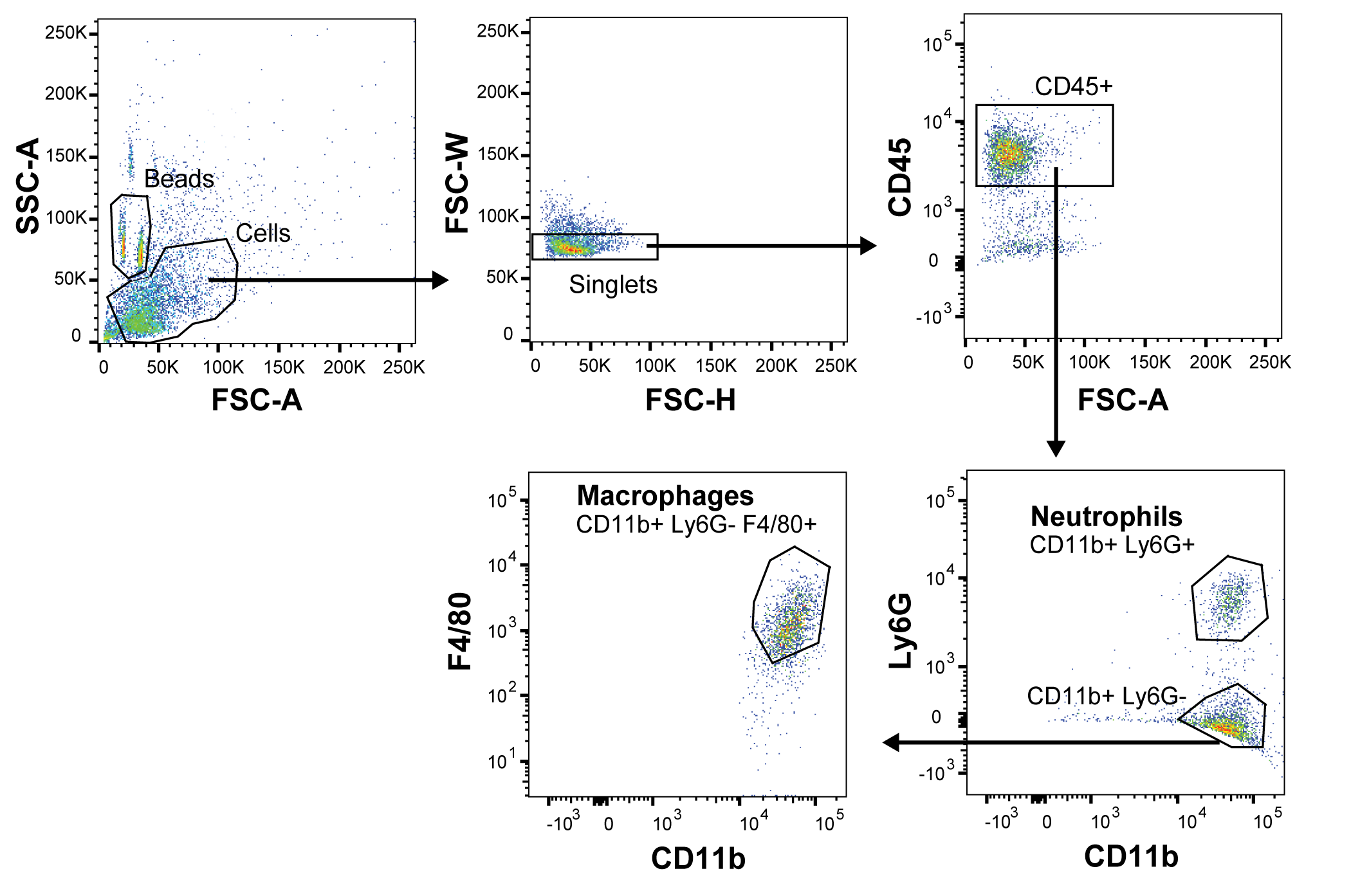


**Supplementary Fig 3: Flow cytometry gating strategy for quantifying immune cell infiltrate in wound homogenates.** Single wound cells were gated from the FSC-A/SSC-A and FSC-A/FSC-W plots. Leukocytes or immune cells were then gated as the CD45+ population, from which Ly6G+CD11b+ (neutrophils) population is gated. The Ly6G- cells were further gated to F4/80+CD11b+ (macrophages) population. To normalize quantification of wound cells, AccuCheck counting beads were gated and quantified from the FSC-A/SSC-A plot as indicated.


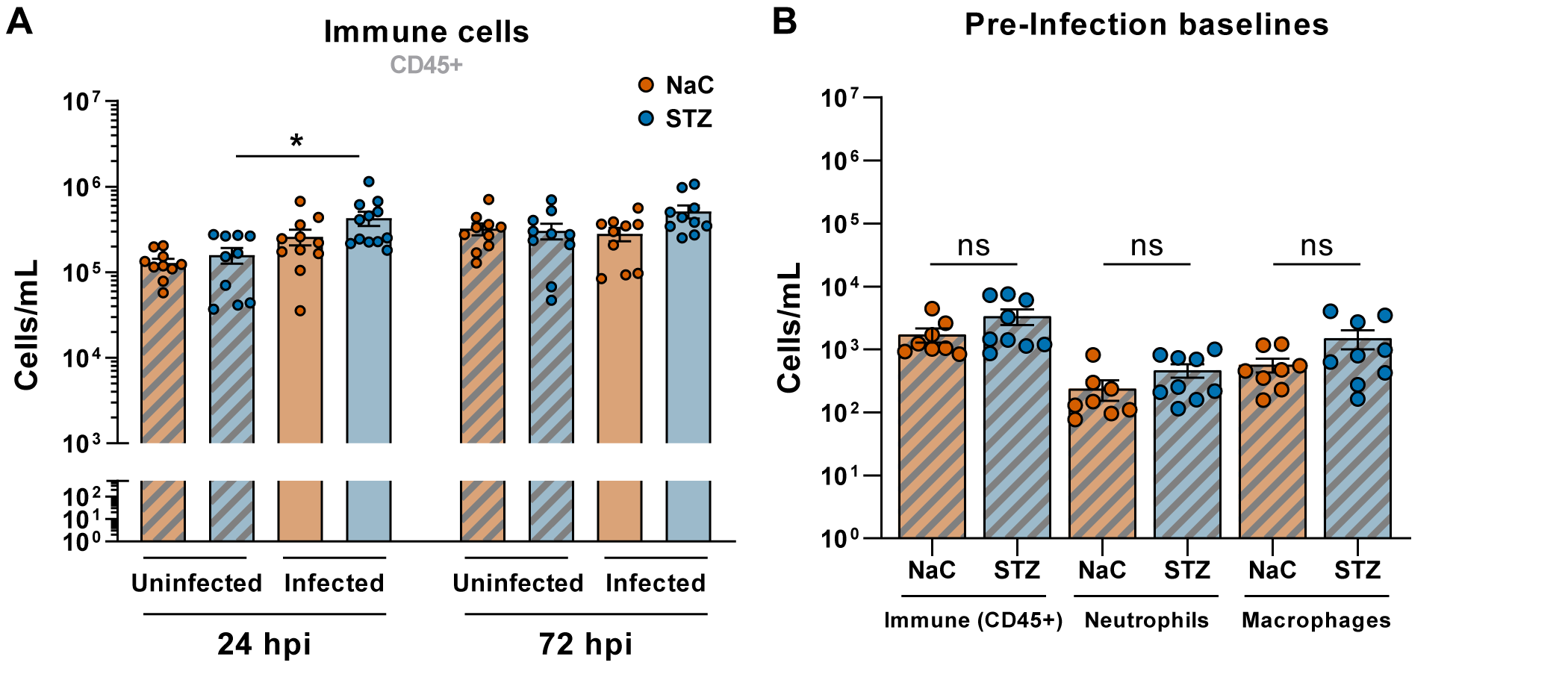


**Supplementary Fig 4: Baseline skin immune-cell populations do not differ between control and diabetic mice.** **(A)** Total numbers of immune cells (CD45+), neutrophils (CD45^+^CD11b+Ly6G+) and macrophages (CD45^+^Ly6G-CD11b^+^F4/80^+^) from control and diabetic mice skin prior to wounding and infection. Bars represent mean ± SEM from n = 8-9 per infection group across 2 independent experiments. Statistical significance was determined by multiple unpaired T-tests. All comparisons were found to be non-significant (ns)  **(B)** Total numbers of immune cells (CD45+) from control and diabetic mice post wounding and infection. Statistical significance between sample groups that differ by one experimental variable was determined by a three-way ANOVA with Šídák’s multiple comparisons test. * p<0.05. All other comparisons were found to be non-significant.


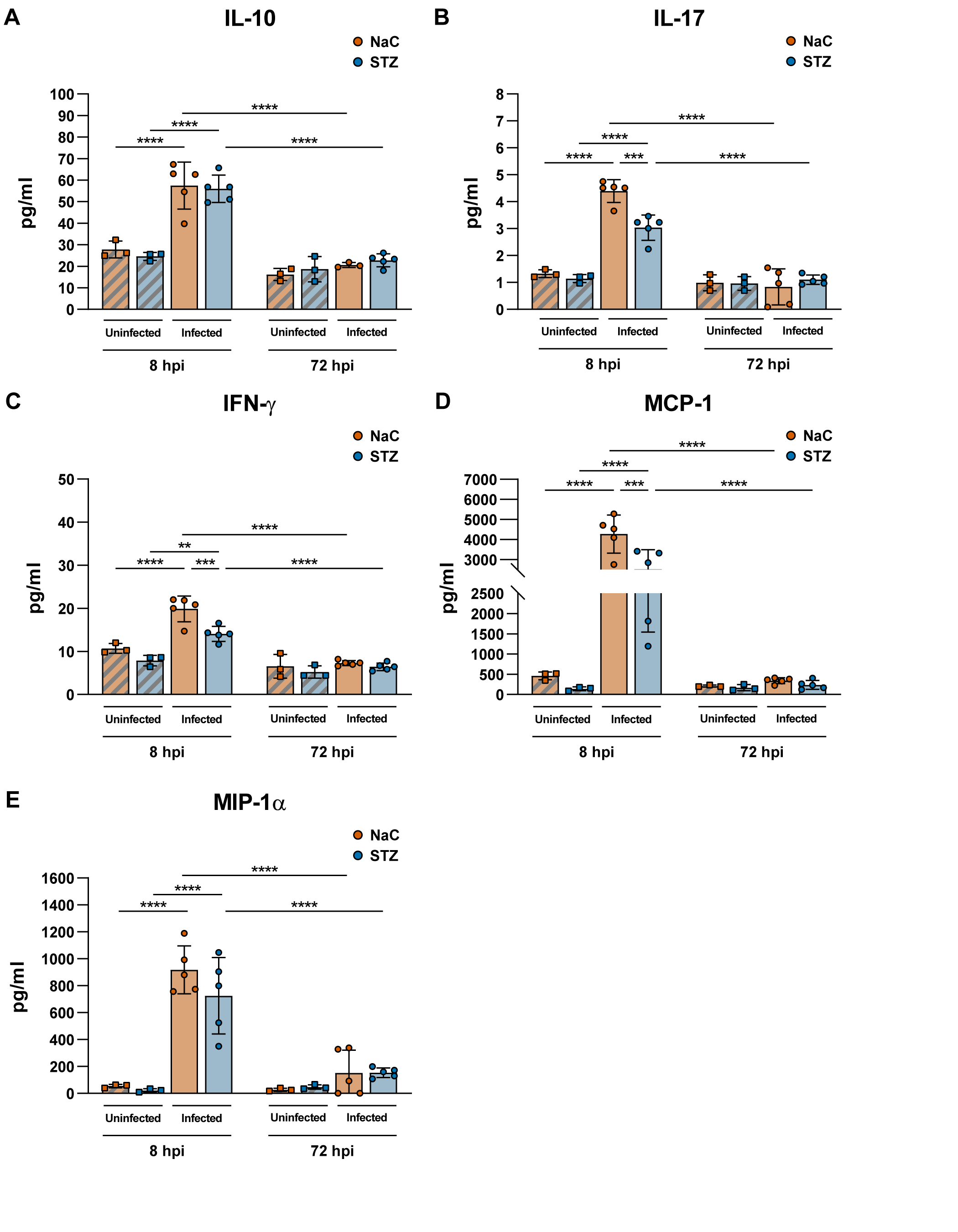


**Supplementary Fig 5: Cytokine levels increased at 8 hours following *E. faecium* infection and diminish by 72 hours.** Cytokine 23-plex assay of **(A)** IL -10, **(B)** IL-17, **(C)** IFN **(D)** MCP-1, **(E)** MIP-1α in various wounds at 8 hpi and 72 hpi. Bars represent mean ± SD from n = 3-5 per infection group from one independent experiment. Statistical significance was determined by 3-way ANOVA with Šídák’s multiple comparisons test.** p<0.01, *** p<0.001 and **** p<0.0001.
